# The precision prevention and therapy of HPV‐related cervical cancer: new concepts and clinical implications

**DOI:** 10.1002/cam4.1501

**Published:** 2018-09-14

**Authors:** Zheng Hu, Ding Ma

**Affiliations:** ^1^ Department of Gynecological oncology The First Affiliated Hospital of Sun Yat‐sen University Zhongshan 2nd Road Yuexiu, Guangzhou Guangdong China; ^2^ Department of Obstetrics and Gynecology Tongji Hospital Tongji Medical College Huazhong University of Science and Technology Wuhan, Hubei 430030 China

**Keywords:** Cervical carcinogenesis, cervical screening, genome editing tools, HPV integration, NGS‐based HPV testing

## Abstract

Cervical cancer is the third most common cancer in women worldwide, with concepts and knowledge about its prevention and treatment evolving rapidly. Human papillomavirus (HPV) has been identified as a major factor that leads to cervical cancer, although HPV infection alone cannot cause the disease. In fact, HPV‐driven cancer is a small probability event because most infections are transient and could be cleared spontaneously by host immune system. With persistent HPV infection, decades are required for progression to cervical cancer. Therefore, this long time window provides golden opportunity for clinical intervention, and the fundament here is to elucidate the carcinogenic pattern and applicable targets during HPV‐host interaction. In this review, we discuss the key factors that contribute to the persistence of HPV and cervical carcinogenesis, emerging new concepts and technologies for cancer interventions, and more urgently, how these concepts and technologies might lead to clinical precision medicine which could provide prediction, prevention, and early treatment for patients.

## Introduction

**Figure 1:**
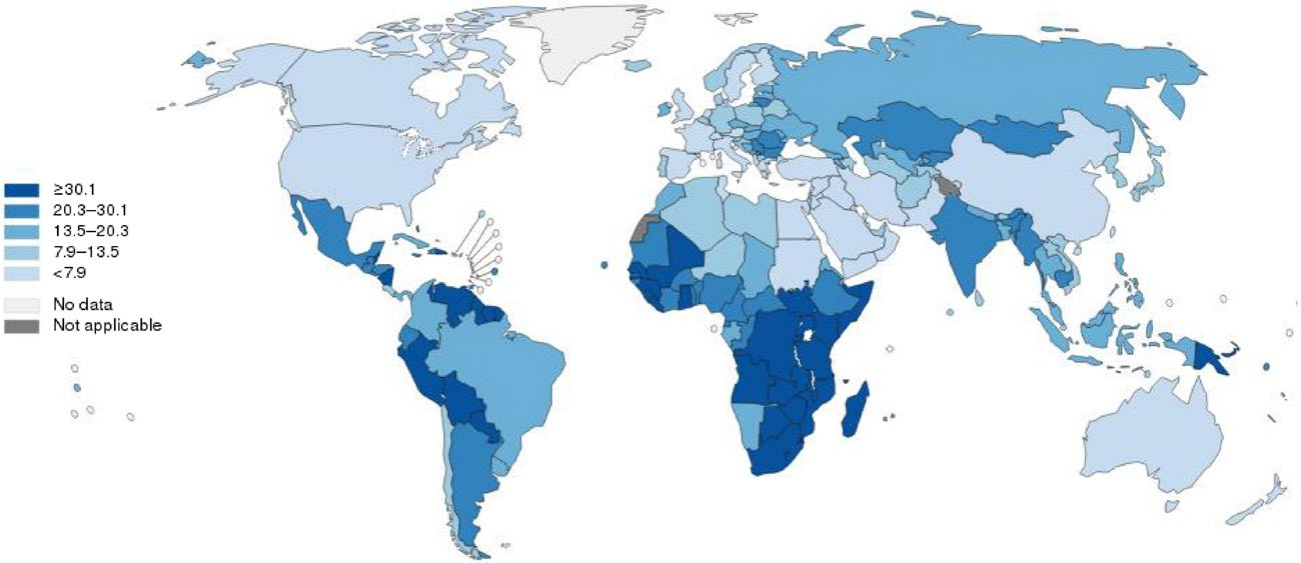
Estimated age‐standardized rates (World) of incidence cases, females, cervical cancer, worldwide in 2012. All rights reserved. The designations employed and the presentation of the material in this publication do not imply the expression of any opinion whatsoever on the part of the World Health Organization/International Agency for Research on Cancer concerning the legal status of any country, territory, city or area or of its authorities, or concerning the delimitation of its frontiers or boundaries. Dotted and dashed lines on maps represent approximate borderlines for which there may not yet be full agreement.

**Figure 2:**
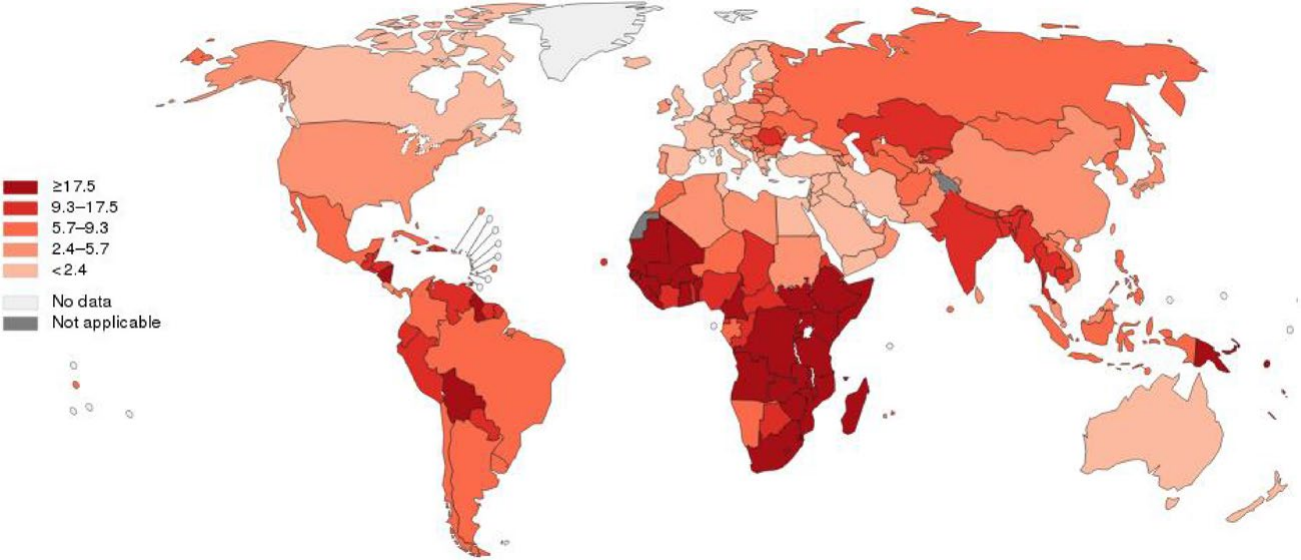
Estimated age‐standardized rates (World) of deaths, females, cervical cancer, worldwide in 2012. All rights reserved. The designations employed and the presentation of the material in this publication do not imply the expression of any opinion whatsoever on the part of the World Health Organization/International Agency for Research on Cancer concerning the legal status of any country, territory, city or area or of its authorities, or concerning the delimitation of its frontiers or boundaries. Dotted and dashed lines on maps represent approximate borderlines for which there may not yet be full agreement.

Cervical cancer remains the third most common cancer in women worldwide, with approximately 529,800 new cases and 275,100 deaths annually 1, 2. Of note, incidences of cervical cancer are disproportionally distributed between developed countries and less‐developed countries 3 (Figure 1). Cervical cancer incidence rates and deaths in well‐developed countries have progressively declined 4 (Figure 2), due to cancer screening programs and HPV vaccination programs funded by huge government budgets 5. In less‐developed countries 6, 7, however, cervical cancer is still one of the most prevalent cancers and the leading cause of cancer deaths in women, many of whom are often diagnosed at an age when they are still raising families. For example in China, a significantly increasing incidence and mortality trend are observed for cervical cancer, especially in young women 7. Cervical cancer has become the second most common female cancer and the third leading cause of cancer deaths in Chinese women aged 15 to 44 years 8. In India, cervical cancer ranks as the second leading cause of cancer in women. An estimated one‐fourth of the world's cervical cancer deaths every year (~77,100) occur in the second most populated country in the world 8, 9. It is worth noting that in recent years, due to the lack of effective prevention/screening methods, incidences of cervical cancer are still increasing in developing countries 3, 10.

HPV infection is recognized as a major causative factor in the development of cervical cancer 11. Epidemiological research shows that in sexually active women, the viral infection rate might be as high as 80% 12. While most of the viral infections are cleared spontaneously by host immunity, a very few persist and eventually cause cancer 13. Thus, cervical cancer is a rare accident in common HPV infections 14. Currently, there is no effective treatment for HPV persistence 15. Prevention of HPV‐related cervical cancer relies on costly HPV vaccines and repeated cervical screenings 16. Although vaccines have shown promising results in recent years 17, the implementation of universal HPV vaccination strategies is expensive for developing countries 5, 18, especially for populous countries such as China and India, and the multivalent vaccines cannot completely cover all the major types of HPV infections in these countries 19. In addition, all commercially available vaccines are prophylactic, and they have no therapeutic effects on existing infections 15. For unvaccinated HPV‐infected patients who are still at risk for cervical cancer, repeated screenings and colposcopy‐directed biopsies are performed. Consequently, these interventions have given rise to potential overtreatment, additional costs, patient anxiety, and adverse effects (e.g., vaginal bleeding and impaired sexual function)^20^, ^21^. Due to the huge economic burden posed by cervical screening and vaccination programs, many women in both developed and developing countries are still unprotected from HPV infections and its related cervical cancers 10.

## Key Factors that Contribute to HPV Persistence and Cervical Carcinogenesis: Host Susceptibilities to Latent HPV Infections and Cervical Cancer

Like many other types of malignancies, cervical cancer is a chronic complex disease caused by a combination of inherited genetic factors and external environmental influences 22, 23. As a major environmental risk factor, HPV infections on their own have been discovered to be insufficient to cause cancer 24. This notion is supported by the phenomenon that 60% of HPV infections regress spontaneously within 1 year and 90% regress within 2 years 25, leaving very few cases that may harbor intrinsic susceptibility for progressing to precancer or cancer. Therefore, efforts to identify inherited genetic risk factors are of great value 26, as they will provide more insights into host–virus interactions 27 as well as an overall etiological understanding of cervical carcinogenesis 22, 28. Obvious evidence of genetic factors contributing to cervical carcinogenesis can be proved by warts, hypogammaglobulinemia, immunodeficiency, and myelokathexis syndrome (WHIM) and hereditary nonpolyposis colorectal cancer (Lynch) syndrome, two autosomal dominant genetic disorder characterized by extensive HPV infection and high risk of cervical cancer 29, 30.

Previous researches on host susceptibility to cervical cancer have focused on the genes that are involved in the human leukocyte antigen (HLA), which is also known as the major histocompatibility complex (MHC) system 31. The system encodes particular antigens to stimulate the multiplication of T helper cells and activates a series of immune reactions to clear that specific antigen 32. The first study that discovered these common variants in the MHC system was conducted in the 1990s, when an increase in relative risk for cervical cancer was seen for DQB1*0303 and DQB1*0604, and a decrease in relative risk was seen for DQB1*0201 and the heterozygote DQB1*0301/*0501 in African‐American women 33. Interestingly, only a few months later, another study conducted with Norwegian patients also report similar susceptible loci DQA1 and DQB1 in the MHC region, with partial but not complete overlap 34. From then on, many more variants of MHC genes were discovered and confirmed to be correlated with cervical cancer in various populations 35, 36, 37, 38, 39. Wu et al. 37.confirmed that the HLA‐II DQB1*0602 allele was significantly increased in HPV16‐infected patients with cervical cancer. Jia et al. 36. found three HLA‐DP SNPs (rs4282438, rs3117027, and rs3077) to be significantly associated with the risk of cervical cancer. The MICA gene, which is located at the MHC I region on chromosome 6p21.33, is another research hotspot for cervical cancer susceptibility. Chen et al. 39 conducted a genomewide association study (GWAS) of cervical cancer in the Swedish population and identified three independently acting loci within the MHC region: rs2516448 (class I region in proximity to MICA), rs9272143 (class II region between HLA‐DRB1 and HLA‐DQA1), and rs3117027 (class II region at HLA‐DPB2). To date, the largest investigation on host susceptibility to cervical cancer has been carried out in the Chinese population 22. The investigation initially included 1364 individuals with cervical cancer (cases) and 3028 female controls, and was followed by two independent validation cohorts (1824 cases and 3808 controls for validation 1 and 2343 cases and 3388 controls for validation 2). Nine significant SNPs were identified within a 180‐kb region that includes HLA‐DPB1/2 and HLA‐DPA1. Together, these association analyses strongly suggest important involvement of HLA alleles or the host innate immune system in the tumorigenesis of cervical cancer. However, unlike cervical cancer, MHC‐associated study for precancer reported rarely met GWAS level and non‐MHC researches need to promote for GWAS significance.

Additional studies also reported the potential roles of common gene variants that are involved in the cellular cycle and apoptosis, cell proliferation and differentiation, DNA repair, and immune responses 40, 41, 42, 43, 44, 45, 46. Nevertheless, as in many other association analyses, evidence of how genetic susceptibility is linked mechanistically to the progression of cervical cancer is still lacking. Therefore, post‐GWAS functional studies (i.e., genetically modified cell lines or mice created by CRISPR) are urgently needed to understand the possible mechanisms of genetic susceptibility to cervical cancer.

## Major Genetic and Epigenetic Events during the Interplay Between HPV and the Host

### HPV variants and cervical cancer

Human papillomaviruses are a big family with the systematic classification of five genera (*α*,* β*,* γ*,* μ*, and *ν*), 48 species, and 206 types 47. The contribution of HPV types to cervical carcinogenesis is different, and responsive classification related to oncogenic degree contain 13 high‐risk (HR) HPV types (16, 18, 31, 33, 35, 39, 45, 51, 52, 56, 58, 59, and 68; IARC Group 1 & 2A), 14 possibly high‐risk types (HPV 5 and 26, 53, 66, 67, 68, 70, 73, 82, 30, 34, 69, 85, and 97; IARC Group 2B), and other low‐risk types (HPV 6, 11, 42, 44, etc.; IARC Group 3)^48^. Among the HR HPV types, HPV16 accounted for more than half of the cervical cancer in the world, while HPV18 making up 16.5% as the second most carcinogenic type 49.

In addition to HPV genotypes, HPV intratypic variants also have epidemiological and oncogenic value in cervical cancer. Based on whole‐genome comparison, HPV16 variants have four major lineages: A, including A1–3 (European), and A4 (Asian) sublineages; B (African 1); C (African 2) and D, including Asian American (AA) and North American (NA). In the same way, HPV18 variants have been defined into three major lineages (A, B, and C) and additional sublineages (A1 to A5 and B1 to B3) with translation [A1 and A2 = AA (Asian Amerindian), A3 to A5 = E (European), and B/C = AFR (African)]^47^.

According to the largest to date oncogenic risk analysis of HPV16 sublineages at Kaiser Permanente Northern California, A4, C, D2, and D3 had higher risk of cervical precancer and cancer compared to extensive A1/A2 sublineages 50. In accordance with this study, a recent meta‐analysis has demonstrated that A lineage accounted for a majority of Asian HPV16 variants, and A4 sublineage was more oncogenic than A1‐3 in China 51. Interestingly, contradictory conclusion was drawn that amino acids of HPV16 E7 shared highly conservation among different sublineages in cervical malignancy by deep sequencing of HPV genome region 52. This study suggests that E7 is the key of HPV16‐related carcinogenesis regardless of viral sublineages. Further investigation on virus–host interaction pattern is also warranted to discover the exact targets for clinical triage and intervention.

In contrast to HPV16, a worldwide study revealed that HPV18 sublinages showed no significant association with cervical cancer risk or histological types 53. However, a previous study in Spain reported that sublineage B had higher oncogenic risk compared to A sublineage but suffered from small sample sizes 54.

Besides, to provide comprehensive guidance for clinical application, it is worth further research that how oncogenic difference among HPV intratypes was influenced by ethnic genome variability, and geographical or behavioral factors.

### HPV integration

Integration of the HPV genome into the host chromosome is a key genetic step in cervical carcinogenesis 55. Numerous studies have shown that integration of HPV normally involves breaking up the open reading frames of viral E1 and E2 regions, resulting in the upregulation of oncogenes E6 and E7 56. E6 and E7 each has multiple cellular targets that promote malignant transformation. For instance, E6 binds and degrades tumor suppressor p53 and pro‐apoptotic protein BAK, thereby increasing host cell resistance to apoptosis and permitting viral DNA replication 11, 57. On the other hand, E7 inhibits tumor suppressor retinoblastoma 1 (RB1) to release E2F transcription factors, and stimulates cyclin‐dependent kinase 2 (CDK2)/cyclin A 58 as well as CDK2/cyclin E complex 59, thus abrogating cell cycle arrest and stimulating proliferation 60.

The first experiment to identify HPV integration in the human genome was published in 1987, when a single copy of HPV16 was detected in the intergenic region between KLF5 and KLF12 on chromosome 13q22 in the SiHa cell line 61. Since then, many PCR‐based detection methods have been developed to explore the genomic interaction between the virus and the host. For instance, ligation‐mediated chain reaction (DIPS‐PCR) 62, 63, 64, 65, 66, 67, 68, 69 and restriction site‐PCR (RS‐PCR) 63, 70, 71, 72, 73 were used to profile HPV integration sites on the DNA level, while the amplification of papillomavirus oncogene transcripts (APOT) assay was used on the RNA level 62, 65, 74, 75, 76, 77, 78, 79, 80. Although these methods were generally labor‐intensive and time‐consuming, the data lay the foundation for the understanding of the HPV integration pattern and its role in cervical carcinogenesis. The major conclusions of the early studies were a) HPV integration sites were distributed randomly on the host genome 81, b) but somehow still show a tendency toward common fragile sites in the genome 73 c) or are more prone to integrate into actively transcribed or easily accessible regions 55. However, the integrated HPV genome was considered only as a disruption of E1/E2 for continued expression of the E6 and E7 oncogenes, but not as having an impact on the integrated genome of the host 62, 74, 82. As evidence accumulated, an emerging point of view that the integrated virus can provide or exert selective advantage over nonintegrated clones began to gain supports 73, 83, 84. Peter et al. showed that HPV integrated at chromosome 8q24 could lead to amplification and overexpression of downstream oncogene MYC 84. This conclusion was later confirmed by the high‐throughput haplotype study of HeLa as well as our genomewide study 85. Another interesting report was published by Schmitz et. al., who demonstrated the correlation between HPV insertional mutagenesis, and loss of CASZ1 and LIPC gene function 86. Generally, the integration sites detected by PCR‐based experiments suffered from bias toward restriction sites in the human genome or the early gene of the HPV genome 28. Although necessary and valuable, the data provided only a limited prospect about the whole picture of HPV integration‐driven tumorigenesis.

With the development of next‐generation sequencing, genomewide profiling of HPV integration sites is becoming feasible and cost‐effective 87. Technical advancements allow researchers to perform highly sensitive HPV integration analysis in larger samples,shedding new light on the underlying mechanisms of HPV integrations. In 2013, Xu et. al. published a novel multiplex strategy named TEN16 for sequence determination of HPV16 DNA integration sites based on next‐generation sequencing, making the concomitant analysis of HPV16 integration sites in a single mixture of about 50 tumor samples possible 88. Based on the experiment, the author suggested that a unique HPV integration breakpoint could serve as an attractive personalized tumor biomarker for prognostic evaluation and treatment of patients with cervical cancer. Akagi et. al. performed WGS on 10 HPV‐positive cancer cell lines and presented a model of ‘‘looping’’ by which HPV integrant‐mediated DNA replication and recombination may result in viral–host DNA concatemers, frequently disrupting genes involved in oncogenesis and amplifying HPV oncogenes E6 and E7 89. Our groups also analyzed HPV integration breakpoints in 26 cervical intraepithelial neoplasia, 104 cervical carcinomas, and five cell lines. Microhomologous sequence between the human and HPV genomes was significantly enriched near the integration breakpoints, indicating that fusion between viral and human DNA may have occurred by microhomology‐mediated DNA repair pathways 28. One year later, Allyson et al. developed a generic and comprehensive capture‐HPV method followed by next‐generation sequencing (NGS) as well 90. Interestingly, the analysis of 72 cervical carcinomas identified five HPV signatures, and cross‐analyses between the HPV signatures and the clinical and virological data revealed unexpected biased representation with respect to the HPV genotype, patient age, and disease outcome, suggesting functional relevance of this new classification.

### DNA mutation of the host genome

In addition to HPV integrations into the human genome of the host genes, somatic mutations of the host genome during HPV‐induced carcinogenesis have also been an important aspect of studying cervical carcinogenesis. DNA mutations analysis plays an important role in identifying differences between cancer tissue and noncancer tissue, and in guiding diagnostic and therapeutic regimens.

To date, the most comprehensive genomic landscape paper was published in Nature Journal; using NGS analysis, it revealed both known and novel high frequent mutations. The authors showed that the common mutations in SCC were EP300 (16%), FBXW7 (15%), PIK3CA (14%), HLA‐B (9%), and p53 (9%) while PIK3CA (16%), ELF3 (13%), KRAS (8%), and CBFB (8%) were in ACC 91. Of note, driver mutations in oncogenes HLA‐B, EP300, and FBXW7 were newly identified in cervical cancers 91.

Besides these novel driver mutations, some important common mutations including the oncogenes PIK3CA, EGFR,KRAS, and the gene suppressor PTEN, p53, STK11, and MAPK genes 92, 93, 94, 95, 96, 97 have already been reported in earlier genetic studies of the polymerase chain reaction (PCR) and Sanger sequencing 96, 98, 99, 100 or spectrometry‐based mutation analysis 101, 102. With the improvement of NGS assay, many researchers also have confirmed the above findings in different cervical cancer samples from different populations 103.

An important application of particular genomic mutations is that they may act as potential early screening biomarkers of cervical cancer. Therefore, numerous studies have explored the somatic mutations spectrum in CINs to cervical cancers and found potential early diagnostic gene mutation markers, such as oncogene EGFR, and PIK3CA, and the gene suppressors TP53 and PTCH1 104, 105, 106. Several other tumor suppressor gene mutations have also been reported in both cervical intraepithelial neoplasms and cervical cancers. LOH11CR2A /EI24/CHEK1 are the receptors of SLIT and are involved in neural axis formation and angiogenesis. In CIN, the mutation frequency of CHEK1, EI24, LOH11CR2A, RASSF1A, PTCH1, and PIK3CA is 28%, 21% 107, 15% 107, 26% 108, 1.5% 97, and 2.4% 105, respectively. Meanwhile, the mutation frequency of CHEK1, RASSF1A, EI24, PIK3CA, and LOH11CR2A increased to 51% 107, 50% 108, 41% 107, 37.1% 105, and 36% 107 in cervical cancers, respectively. Chakraborty mentioned that the deletion of PTCH1 gene was 42% in FIGO stage I/II 97 and 46% in stage III/IV 97. Besides the common genomic mutation event, Goia‐Ruşanu et al. also found an interesting phenomenon, in that the D‐loop region mutation in mitochondrial DNA also existed differently in different cytology status 109. No mutations were detected in either normal or ASCUS cytology state, but an increasing rate of mutation detection was found in LSIL, HSIL, and SCC cases 109.

Studies also showed that gene mutations can have an impact on the prognosis of cervical cancer. CHEK1, EI24, PTCH1, and ATM belong to the PI3/PI4‐kinase family, and the tumor suppressor gene CADM1 is a predictor of the worst prognosis 97, 107, 110. ATM and CADM1 can predict early invasiveness 110. The PIK3CA mutation can decrease the distant metastasis rate 111.PIK3CA mutation in 771 Chinese patients treated with surgery‐based comprehensive therapy significantly improved the 3‐year relapse‐free patient survival rate 111. Moreover, studies have also illustrated that patients with KRAS mutations experienced an obvious declining recurrence‐free survival rate 112.

### DNA methylation of cervical carcinogenesis

Another prevalent epigenetic mechanism during HPV‐induced carcinogenesis is DNA methylation, which is a methyl (‐CH3) covalent addition to cytosine in the DNA sequence known as CpG dinucleotide 3. CpG can accumulate in the CpG island, which is a CpG‐rich sequence that is often located in the gene promoter 113. During the progression of a malignancy, local hypermethylation of the CpG islands in the promoter regions of the downstream tumor suppressor genes can lead to their downregulated expression 114.

As exogenous pathogen, HPV epigenomic pattern has been thought to have prominent significance. Although the CpG islands on HPV genome are underrepresented, high density of CpG sites and characterized difference in E2/E4 region and alpha 9/7 clades still exist, indicating the underlying value of HPV DNA methylation mechanism 115, 116.

To identify specific methylation alteration of HPV DNA, bisulfite‐based methylation‐specific PCR (MS‐PCR) and sequencing were used and demonstrated hypermethylation of LCR region of HPV 16 correlated with oncogenic progression in cell lines and cervical lesion 117. With pyrosequencing method, cervical carcinogenesis was found related to the methylation of L1, L2, and E2/E4 region in HPV16 genome 118. Another whole‐genomewide pyrosequencing of HPV18, HPV31, and HPV45 revealed significantly elevated DNA methylation level in E2, L1, and L2 region in cervical intraepithelial neoplasia grade 3 (CIN3 + ) than transient infection 119. As this research suggested, HPV methylation may serve as potential malignancy biomarker to distinguish the transforming infection. With NGS‐based analysis of HPV16 CpG methyl‐haplotypes, a study testified the diagnostic effect of hypermethylation of E2, L1, and L2 region for CIN 3 +  patients 120. Recently, a targeting and methylation deep sequencing method reported that global HPV methylation level detects >95% ICC in HPV16 positive samples, specifically, with L1 as best marker for 13 HPV types and E1 as novel marker associated with HPV 16 121. However, the mechanism of HPV DNA methylation as biased molecular for HPV persistence and cervical cancer progression is not fully understood, which also strongly suggests further dedication to virus–host interaction research.

Besides, high‐risk HPV E6 and E7 are directly related to functions of DNMTs(DNA methyltransferase), which are the key enzymes responsible for DNA methylation 122. Further research of HPV‐related methylation showed downregulation of E6‐ and E7‐reduced methylation of tumor suppressor genes, thereby reversing the malignant phenotype of cervical cancer cells 123. This evidence indicates that HPV oncogenes are related to host gene methylation.

In accordance with the data, DNA methylation was found to be a common event in cervical carcinogenesis. The level of methylation was positively correlated with the severity of both CINs and cervical cancer. Hesselink and Bierkens developed an objective nonmorphological molecular method of CADM1/MAL methylation as a cervical cancer screening marker with high sensitivity (ranging from 100% to 60.5%) for CINIII and patients with cervical cancer 124, 125. Meanwhile, using a multiplex quantitative methylation‐specific PCR, a recent study found a good concordance (78%) of CADM1/MAL methylation in biopsy results. The positive detection rates of gene methylation were 5.5% in normal biopsies, 63.3% in CIN3, and 100% in cervical cancer, respectively 126. In two independent cohorts of patients with cervical cancer (*n *= 149, *n *= 121), using Illumina 450K methylation arrays, Lando indicated that promoter methylation events at 3p11‐p14 could become a positive progression marker and a prognostic marker for cervical cancer 127. Further studies on the functional research of these genes, including tumor inducer gene STK31, tumor suppressor PTCH1, and PTPRR indicated these methylation markers could be used in cervical screening to predict poor prognosis of the precancerous lesions 97, 128, 129. In an analysis of promoter methylation of the PTEN gene using methylation‐specific PCR, the data showed that methylation of the PTEN promoter was detected in 61% of the specimens. There was a positive correlation between PTEN methylation and the FIGO stage 130. Low frequency (14–16%) of PTCH1 methylation was seen in the asymptomatic exfoliated cervical cells and in the normal epithelium adjacent to the tumor, followed by a significant increase in CIN (31%) in stage I/II (57%) and comparable CIN in stage III/IV (58%) 97.The methylation frequency is also different in pathological classification. The hypermethylation of the candidate tumor suppressor gene, RASSF1A, was detected in 30% of SCC and 12% of AC, while it was absent in all normal tissues 131.

All above showed that altered DNA methylation pattern of high‐risk HPV types and host genome has the potential application in the risk‐based cervical cancer screening and diagnose, suggesting further study involving large‐scale population experiment to test the effect.

### Molecular subgroups of cervical cancer

Along with accumulation NGS data of cervical cancer, molecular characterization of different levels from genome to metabolism becomes gradually clear. However, achieving management of NGS data by integrative analysis proposes huge challenge but inevitable task to classify virus‐induced carcinogenetic subgroups by comprehensive molecular features, which is the guidance of ultimate clinical application of unbiased NGS strategy.

Recently, the Cancer Genome Atlas Research Network conducted a multi‐omic analysis of invasive cervical cancer on 228 extended samples 132. It combines multiple platforms including whole‐genome sequencing (WGS), whole‐exome sequencing (WES), RNA‐seq, microRNA‐sequencing (miRNA‐seq), DNA methylation profiling, and reverse phase protein array (RPPA) to classify molecular subtypes of cervical cancers 132. Generally, this research defines three clusters keratin‐low squamous, keratin‐high squamous, and adenocarcinoma rich with different molecular features mainly based on mRNA expression analysis 133. And for the first time, this study predicts endometrial‐like (UCEC‐like) cancers with ARID1A, KRAS and PTEN mutated, low copy number, low CpG island hypermethylated (CIMP‐low), and RPPA hormone‐associated features 132. As for high‐risk HPV‐related carcinogenesis, it first reports type‐associated molecular signature except integration 133.

Of note, the most important value of this article is the intention of serving clinical translation and reasoning. By structural variation analysis, the TCGA researchers identified BCAR4, CD274 (PD‐L1), and PDCD1LG2 (PD‐L2) linked rearrangement events. PD‐L1 and PD‐L2 are two important immune checkpoints as well as promising immunotherapy targets 132. Furthermore, the integrative analysis revealed >70% genomic alterations in either P13K‐MAPK or TGF*β* pathway, also indicating potential therapeutic agents 132. Besides, other contribution of this paper includes five new hypermutated genes and strong correlation of APOBEC somatic mutation 132.

In summary, with epigenomic and genomic data on cervical cancer and precancers being accumulated, a fundamental ground for the molecular classification of any HPV‐associated carcinogenesis has been established, as well as personalized biomarkers for each stage 134. However, we need further research to better understand the molecular subclassification of cervical lesions and, more important, develop precision medicine for patient diagnose and treatment 135.

## New Concepts and Technologies for Early Prevention and Treatment of Cervical Cancer

### Current primary and secondary prevention of cervical cancer

Pap cytology has brought up as standard secondary cervical cancer screening for more than half a century, with standardized evaluation Bethesda System established in 1988 and other modified technique like liquid‐based cytology (LBC) and process automation developed in 2000s 136, 137, 138. Undoubtedly, nationwide coverage and high‐quality Pap test significantly reduced at most 80% mortality of the cervical cancer in developed countries (International Agency for Research on Cancer, released on 3 May, 2004). At the meantime, as the role of high‐risk HPV in cervical carcinogenesis fully understood and existence detected in exploited cervical cell sample in 1990s, molecular test of HPV DNA was gradually acknowledged as more sensitivity tool than Pap test alone. At first, HPV test was advised for alternative triage of patients with abnormal cytology results before 2003. Along with the increasing attachment of HPV infection especially high‐risk HPV types and mature of detection method, alternative cotesting of HPV and Pap smear was introduced as well as HPV genotyping 139. However, the positive effect of HPV test alone was not affirmed, which stimulated amounts of clinical trials such as Indian trial in 2009 and Addressing the Need for Advanced HPV Diagnostics (ATHENA) trial in 2014 to encourage approval of HPV test alone as primary screening strategy 140, 141. At the same year of ATHENA trial finished in the United States, The Food and Drug Administration (FDA) approved the first HPV test for primary cervical cancer screening 142. In 2015, an interim guideline, later a new cervical cancer screening and prevention *Practice Bulletin* published, recommended HPV genotyping test alone as primary screening combined with cytology triage, which has been implemented in some developed countries 143, 144.

However, given that HPV test is accompanied by high false positive, the unavoidable increasing consequences of referral to colposcopy would bring psychological side effect and downstream overdiagnosis and overtreatment. Besides, in developing country where >80% cervical cancer incidence was observed, the screening strategy was not performed the best effect due to complex reasons including socioeconomic disparity, low screening coverage and limited funding of government, high personal cost and lack of related health knowledge and crucially, low quality of screening services, and unsound follow‐up management 145, 146. On the other hand, in developed countries, repeated screening program has posed huge economic burdens on the government budget and psychosomatic pressure on healthy women in the program. All above suggest that successful as the current screening strategy is, improvement still needs for the risk‐stratified screening program.

Novel screening strategies of cervical cancer in development focus on the utility of biomarkers and other molecular tests on account of the objectivity and reliability, including p16/ki67, E6/E7 mRNAs, microRNAs, and methylation of HPV and host genome 147. However, none of these tests could predict the potential population that will have persistent HPV infection and progress to cervical cancer, which is personalized associated with host susceptibility, environmental factors, and behavioral pattern besides HPV infection. This arises many related researches to improve current screening efficacy. Instead of one‐size‐fits‐all program, a risk stratification model with bioinformatic methods was made to take full advantage of screening history of patients 148. Besides, a previous paper reported that multiple regression assay and simple artificial learning algorithm achieved high sensitivity and specificity effect to predict cervical cancer 149. These studies indicate that research involving large‐scale population, dynamic bioinformatic tools, and comprehensive information is worthy of further conduction to create a personalized risk prediction model of HPV persistence and cervical carcinogenesis.

Except for HPV test, followed great invention was prophylactic HPV vaccine approved in 2000s. The FDA has approved bivalent vaccine Cervarix against HR‐HPV16/18, responsible for 70% cervical cancer, quadrivalent vaccine Gardasil preventing infection with HPV6/11 added and 9‐valent vaccine Gardasil9 against additional five types 31, 33, 45, 52, and 58. The primary prevention of HPV‐related cervical cancer with HPV vaccine was strikingly promising and many countries have in succession initiated the national immunization program for girls aged 9‐25 before sexual behavior onset. However, with the vaccinated girls reaching screening age, the current prevention and screening guideline is not fully ready for the differentiation between who has been HPV‐vaccinated and who has not. A recent study using model‐based analysis suggested that for HPV‐vaccinated women, the screening should start later, take place less frequently and involve primary HPV testing rather than cytology, much different from current guideline routines 150. Unanimously, an Italian study reported that the tailored screening protocol for HPV‐vaccinated women should start at 30 with HPV test and have longer intervals of rescreening for HPV‐negative results, which still needed further research to determine 151.

### Current HPV testing methods for cervical screening

As persistent infection with high‐risk types of human papillomavirus (HPV) is a necessary condition for cervical carcinogenesis, in recent years HPV detection has gradually become the primary screening method for cervical cancer, instead of cervical cytology. The development of HPV tests is still growing rapidly. To date, at least 193 different HPV tests are available for the detection of HPV in cervical specimens 152. However, of these tests, only 110 (57%) have been cited more than once in the literature, and only 69 (35.7%) have been analyzed or clinically evaluated in publication 152. Solid clinical data are urgently needed to evaluate the efficacy of new techniques.

The Hybrid Capture 2 (HC2) HPV DNA test has been widely adopted to determine HPV infections and to detect HSIL and cervical cancer. Generally, sensitivities for detection of CIN2 or greater ranged from 84.9% to 100%, and specificities ranged from 69.5% to 95.8% 153. The Cervista HPV HR test was approved by the FDA in 2009. A number of clinical trials reported that the Cervista HPV HR test could more accurately detect high‐risk HPV compared to the HC2 test and may have the advantage to lower the cross‐reactivity to other HPV types 154, 155, 156. Two years later, the Cobas 4800 HPV test was FDA approved in 2011. Clinical studies showed that the Cobas test's sensitivity was comparable to that of the HC2 test, but the Cobas test had improved specificity because it has a lower level of cross‐reactivity with other low‐risk HPV types 157, 158, 159. However, false‐negative results may occur because the L1 gene is lost when the virus integrates into the host genome in a substantial number of patients 160.

Recently, detection of the HPV E6/E7 oncogene mRNA has become an alternative to the HPV DNA test. Studies show that little expression of E6/E7 mRNA may be detected in transient infections, but in persistent infections, E6/E7 mRNA is overexpressed. Therefore, detection of upregulated expression of E6/E7 mRNA can be directly related to disease progression and may further reduce the number of colposcopy referrals 161.

### Potential future for NGS‐based HPV testing

**Figure 3:**
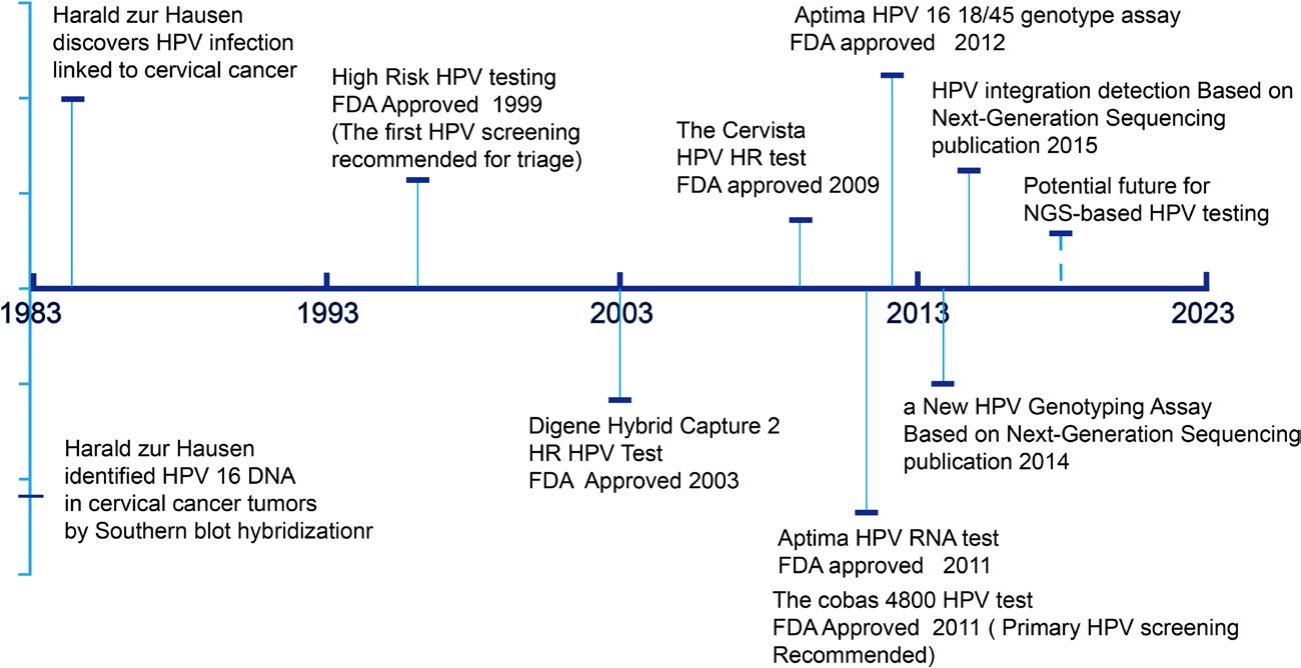
HPV testing methods for cervical screening timeline. Brief overview of screening practice based on HPV changes and related discoveries. FDA, Food and Drug Administration; HPV, human papillomavirus.

To date, there are numerous HPV tests, including PCR‐based MY09/11 and CPI/II systems 162, the hybridization‐based SPF LiPA method; signal‐amplification assays (Hybrid Capture 2 and Cervista); and nucleic acid‐based amplification‐like microarray and real‐time PCR‐based methods (the Cobas 4800 real‐time test)^162^, ^163^, ^164^ (Figure 3). These techniques seemed to perform well, but still may have many limitations, for example difficulty in detecting minor and low‐abundance HPV types, or a mixture of coinfections 165, 166. More important, this generation of screening methods could not detect the integration status of the virus, which is a key genetic event in the development and progression of cervical cancer. In recent years and in the near future, emerging new technologies of NGS have the potential to overcome such limitations. There have already been investigations using TEN16 or HIVID methodology to determine coinfection among the HPV types as well as HPV integration sites in the human genome 28, 88, 167, 168, 169. Therefore, detection of HPV integration into the human genome by NGS as a means of screening for cervical cancer is becoming a new strategy based on HPV screening. Many studies have confirmed that HPV integration and CIN progression are also closely related 3, 4, 5. HPV integration often occurs in low‐grade cervical intraepithelial lesions in the early stage and then progressing to high‐grade intraepithelial lesions 3. The rate of HPV integration is increasing dramatically from CIN to cervical cancer, which provides us with a potential predictor of disease progression. NGS‐based HPV testing also can be more specific than hybridization‐based methods and may be considered the reference standard for genotyping. For example, based on sequencing of the HPV L1 gene, the data will allow us to achieve a better specificity of viral genotyping and may lead to the discovery of new HPV types 170, 171. Therefore, future possible applications of NGS‐based HPV screening include (i) determination of HPV integration sites in the human genome for risk stratification, (ii) precise detection of HPV types in cervical lesions, (iii) epidemiological monitoring of both low‐risk and high‐risk HPV type distribution, and (iv) discovery of new HPV genotypes.

However, data of NGS‐based HPV testing are still lacking, and the technology clearly has both advantages and disadvantages. First, with the knowledge that the NGS‐based HPV test is highly sensitive, the data of this method must be interpreted with care, because high sensitivity also means a high false‐positive rate and possible overtreatment of patients. Second, costs and turnaround time are the major problems with diagnostic methods based on NGS technologies 172; however, with the development of new techniques, both the price and the length of time needed to perform NGS‐based tests are dropping drastically. And as for the interpretation, there is emerging software to detect infected virus types and more importantly, virus integration status and specific region based on chimeric reads and other algorithms. Although software could obtain high sensitivity and specificity detection effect in diverse NGS data of virus‐related (HBV, HPV, and EBV) cancer, including whole‐genome sequencing, targeted sequencing, and RNA‐seq 167, 173, 174, 175, 176, 177, 178, 179, there is still great challenge in applying to clinical practice. Given the complex variation induced by HPV integration, most software has common problem in standardization under different circumstance and therefore still relies on the PCR‐based Sanger sequencing to testify the accuracy. Importantly, for most users especially clinician, who do not possess bioinformatic knowledge and skills, the application is extremely restricted and accordingly the transforming period will be much longer.

Overall, it is quite possible NGS‐based assay will become even more cost‐effective than the classical HPV testing assays such as Cobas 4800. Nevertheless, full automation and standardization of protocols for library preparation and sequencing as well as user‐friendly and mature interpretation tool are urgently required for the implementation of this technology in routine diagnostics 180.

### Immunotherapy of cervical cancer

Along with the prevalence of HPV vaccination and cervical cancer screening, the treatment of early stage cervical disease also advanced, together will continue decreasing the incidence and mortality of cervical cancer 181. However, for those patients who have invasive, advanced‐stage or recurrent cervical cancers. Traditional treatment including systemic chemotherapy, surgery, and radiotherapy offers limited choices. These patients still suffer the great pain and insignificant survival rates brought by treatment and do not benefit from the research outcomes of interplay patterns between host and HPV during malignant progression. Although HPV is the key factor for causing almost all cervical cancers, the infection alone is not sufficient for malignancy of decades progression that additional tumor‐promoting steps needed, especially the evident evasion of immune surveillance 182. Therefore, new paradigm such as immunotherapy provides promising opportunity for the treatment of HPV‐driven carcinogenesis.

The current immunotherapy methodology with clinical trials undergoing or accomplished includes therapeutic vaccine (NCT02164461, NCT02291055, NCT01304524, NCT02172911, NCT02128126, NCT00988559, and NCT0078164), targeted antibodies (NCT01778439, NCT00803062, and NCT01281852), T‐cell immune checkpoint inhibitor (NCT01711515, NCT01693783, NCT0147121, NCT01714739, NCT02205333, and NCT01693562), and adoptive T‐cell transfer (ACT; NCT01585428).

As a paper commented, the targeted therapies of cervical cancer revealed encouraging though limited effect 133. For example, anti‐VEGF antibody bevacizumab combined to chemotherapy improved 3.7 months in median overall survival of patients with advanced‐stage cervical cancer 183. So are clinical trials of other algorithms. Bacteria‐vector vaccine ADXS11‐001 without cisplatin was found no different survival outcome and tumor response but a safety profile compared to with cisplatin 184.A phaseIIstudy of ACT clinical trial has shown complete regression on two of nine metastatic cervical cancer patients but no overall survival influence reported, indicating that further investigation is warranted promisingly 185. In addition, T‐cell immune checkpoint inhibitor such as pembrolizumab targeting the popular PD‐1‐PD‐L1 axis was found tolerant and promising in PD‐L1‐positive recurrent or metastatic cervical squamous cell cancer 186.

Overall, there are still modified immunotherapy researches and clinical trials in progress hopefully to improve the survival of the patients who have limited choices.

## Genome Editing Tools for Treatment of HPV Infections and Their Related Cervical Lesions

**Figure 4:**
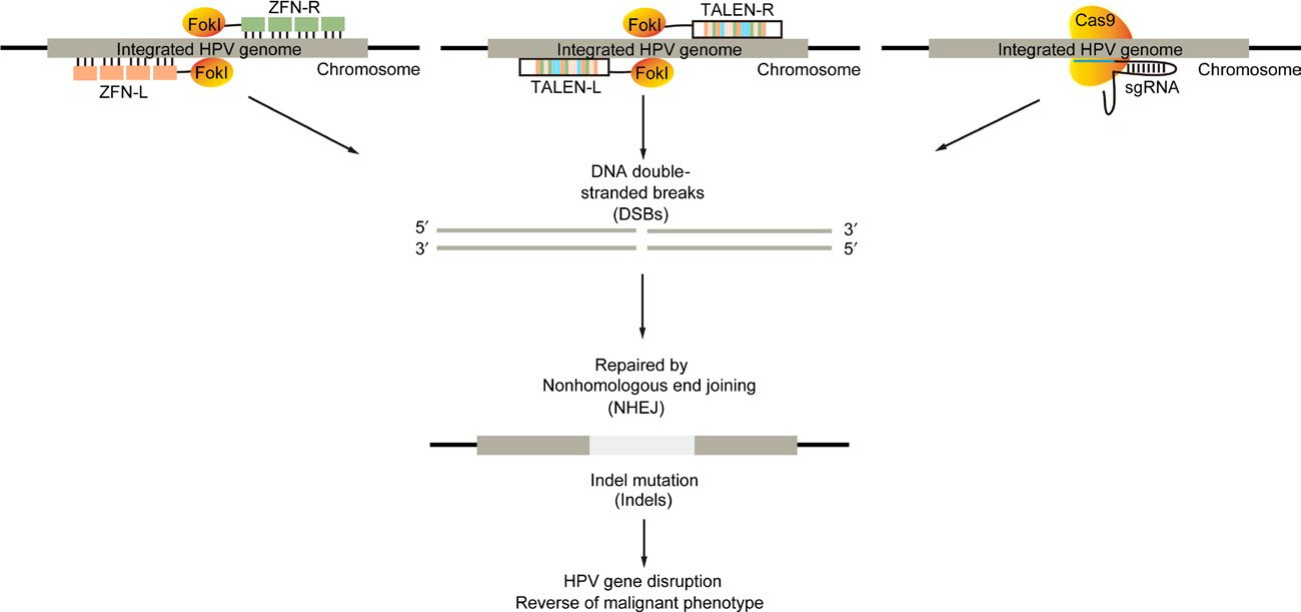
Genome editing as a therapeutic strategy for HPV infections. Diagram of HPV genome editing using ZFNs, TALENs, and CRISPR/Cas9.

Currently, there is no effective treatment for HPV persistence. Previous researchers (including those from our laboratory) have shown that targeting HPV E6/E7 mRNAs with siRNA could effectively knock down their expression and induce apoptotic cell death in HPV‐positive cell lines. However, siRNAs only block HPV E6/E7 mRNAs temporarily, and they do not attack HPV DNA in the nuclei, which serves as a store of escape mutants that cause resistance to siRNA application. In recent years, artificially engineered genome editing techniques such as zinc finger nucleases (ZFNs), Tal‐effector nucleases (TALENS), and the RNA‐guided engineered nucleases (RGENs or CRISPR/Cas9) were used to cleave the specific DNA sequence of HPV. Generally, the double‐stranded DNA breaks (DSBs) introduced by these custom‐engineered endonucleases should trigger DNA repair pathways (NHEJ repair pathway in most of the cases), resulting in the disruption of target viral oncogenes and the elimination of HPV infections (Figure 4).

In the early stage of development of HPV‐targeted genome editing tools, ZFNs were designed to disrupt the E6 or E7 genes of the high‐risk HPV types 16 or 18. These reports showed ZFNs could disrupt HPV genomes in HPV‐positive cell lines and in vitro culture models. Of note, one study demonstrated that injected ZFNs may even inhibit growth of HPV‐positive tumor xenografts, implicating their potential as anticancer drugs. However, ZFNs also have some limitations. First, construction of ZFNs is very labor‐intensive and may be hard to replicate in laboratories that do not have sophisticated skills molecular engineering skills. Second, the specificity of ZFNs varies greatly in different studies. Generally, the lengths of the recognition sequences are determined by the number of zinc fingers for the corresponding nuclease (often 18–24 nucleotides for 6 to 8 fingers). Although increasing the number of zinc fingers would improve their specificity comparable to that of the TALENs, extensive experiments would be required to screen the best combinations of ZFN monomers with different numbers of zinc fingers 187.

Another comprehensive study has demonstrated that TALENs as well as ZFNs can also be used as a therapeutic strategy to treat HPV‐related cervical lesions. In the study, the author achieved comparable or better efficacy to cleave E6 and E7 genes of HPV‐16 and HPV‐18 using artificially engineered TALENs. Interestingly, through topical application of TALENs directly onto the cervix of the HPV‐driven malignant phenotype of K14‐HPV16 transgenic mice, cervical intraepithelial neoplasms regressed to normal cervical tissues. Mutations of the E7 gene and reduction in the HPV16 DNA load were further confirmed. One potential advantage of TALEN is that its longer DNA‐binding sequence (approximately 30–40 nucleotides or more) may determine its improved specificity over clustered regularly interspaced short palindromic repeat (CRISPR) RNA‐guided nuclease 187, ZFN, and siRNAs. On the other hand, the longer DNA‐binding sequence of TALENs, as well as their larger backbone, could be difficult to pack into vehicles such as AAV or lentiviral delivery systems.

With the emergence of CRISPR, this widely adopted technique has also been developed to target the E6 or E7 genes of HPV types 6, 11, 16, or 18. The antiviral efficacy of CRISPR/Cas9 has been shown in a number of in vitro cell culture models and also in HPV‐positive tumor xenograft models. Disruption of HPV E6 and E7 led to downregulation of their corresponding viral proteins and resulted in restoration of the tumor suppressor p53 and pRb. The primary concern of the CRISPR technique is its off‐target effects when it is applied in human subjects. The specificity of the newly developed and RNA‐guided CRISPR endonuclease is mainly determined by the protospacer adjacent motif (three nucleotides) and seed sequences (approximately 12 nucleotides), which are located at the 3′‐end of its recognition sequence GN20GG 188. The relatively short length of the CRISPR recognition sequence (approximately 14 nucleotides) makes the endonuclease more prone to produce undesired, off‐target mutations in the human genome, thus limiting its further use in therapeutic applications 189. Nevertheless, the specificity of the CRISPR system was recently improved by the paired RNA‐guided, double‐nicking strategy (approximately 28 nucleotides), or the modified high‐fidelity versions of CAS9 190.

Genome editing‐based antiviral therapy may play valuable roles in HPV‐related cervical carcinogenesis. If this therapy is appropriately combined with HPV testing, patients in precancerous stages, such as persistent HPV infections and their related CINs, would be able to take HPV testing first to determine the HPV subtype(s) and then select the corresponding type‐specific genome editing tools to treat the appropriate HPV infection(s) and their related precancerous diseases. These patients would benefit from this new “screen‐and‐immediately‐treat” strategy, instead of potential overtreatment including repeated screenings, colposcopy‐directed biopsies, and cold knife conization. Consequently, additional costs, patient anxiety, and adverse effects that can result from repeated screenings, colposcopy‐directed biopsies, and cold knife conization (e.g., vaginal bleeding and cervical insufficiency) could be avoided.

In the future, more study still needs to be performed. When applied to human subjects, the length of time that the complexes need to stay resident in the vagina must be further determined; to extend this time, polymer‐complexed artificially engineered nucleases could be directly applied to the anogenital tract using devices such as the CerviPrep drug delivery system 191 or they could be formulated into vehicles suitable for anogenital tract retention (e.g., suppository, gel, or cream)^192^. In addition, physiological changes of the vagina and cervix that occur with the menstrual cycle, particularly the pH value and vaginal fluid, should also be taken into consideration when artificially engineered nucleases are delivered intravaginally 193.

## Future Directions and Conclusion

With the emerging new concepts and technologies for cancer interventions, the precise prevention, diagnosis, and treatment of cervical are not only necessary, but now 194. The clarification of the molecular mechanism underlying HPV persistence and related cervical cancer will help us to predict the prognosis of patients with HPV infections at an earlier stage. Molecular classification based on HPV integration and genetic profiling may also translate into the precision medicine that allows clinicians to focus medical recourses more on high‐risk patients whose diseases are genuinely progressing, greatly reducing the psychological and economic burdens of the cervical screening programs and HPV vaccination programs in the future.

## Online Method

Figures 1 and 2 were made online: http://gco.iarc.fr/today/online-analysis-map?mode=cancer&mode_population=continents&population=900&sex=2&cancer=16&type=1&statistic=0&prevalence=0&color_palette=default&projection=natural-earth#modalTakeATourVideo

## Conflict of Interest

None declared.
